# 2,7-Carbazole Derived Organoboron Compounds: Synthesis and Molecular Fluorescence

**DOI:** 10.3389/fchem.2021.754298

**Published:** 2021-10-22

**Authors:** Minhui Chen, Juan Wei, Yufeng Zhang, Lin Wu, Leibo Tan, Shanglong Shi, Junqing Shi, Lei Ji

**Affiliations:** Frontiers Science Center for Flexible Electronics, Xi’an Institute of Flexible Electronics (IFE) and Xi’an Institute of Biomedical Materials and Engineering, Northwestern Polytechnical University, Xi’an, China

**Keywords:** organoboron compound, carbazole (Cz), highly luminescence, triarylboranes, donor-acceptor

## Abstract

Triarylboranes have drawn much attention in OLEDs owing to their remarkable solid-state luminescence properties. Here two new A-D-A type compounds, 2,7-bis(dimesitylboryl)-N-ethyl-carbazole (BCz) using triarylborane as electron acceptor and carbazole as electron donor while 2,7-bis((4-(dimesitylboryl)phenyl)ethynyl)-9-ethyl-carbazole (BPACz) using phenylacetylene as extra conjugated bridge, have been synthesized and their photoluminescence related properties in various states have been investigated both experimentally and theoretically. Both compounds show blue emission with high quantum yields, being potential candidates for blue OLED materials.

## Introduction

Luminescent organic *π*-conjugated molecules play an important role in modern materials science, life and analytical science, such as optoelectronics, sensors, lasers, smart multi-responsive materials, and bioprobes (Anthony, 2006; Chi et al., 2012; Jun-Jie et al., 2020; Wu et al., 2021). Recently, special emphasis has been paid to organoboron compounds with unique photophysical properties and leading to versatile applications (Noda and Shirota, 1998; Hung and Chen, 2002).

Triarylborane possesses a sp^2^ (Chi et al., 2012)-hybridized boron, where an empty *p*_z_ orbital remaining allows intramolecular electron delocalization in a trigonal planar geometry (Brown and Dodson, 1957), which gives relatively lower unoccupied molecular orbital (LUMO) levels to enhance the electron affinity and tunable highest occupied molecular orbital (HOMO) levels with electron donor groups (Hudson and Wang, 2009; Wade et al., 2010) to produce low energy intramolecular charge transfer (ICT) bands upon photoexcitation (Sakuda et al., 2010), being beneficial for optoelectronics such as organic light-emitting diodes (OLEDs). In the past two decades, after the pioneering research of Yasuhiko and Shirota et al. (Noda and Shirota, 1998; Shirota et al., 2000), triarylboranes (Frieder, 2006; Jäkle, 2010; Turkoglu et al., 2017; Berger et al., 2021), B bonding with three aryl rings that are usually strongly luminescent, have been developed rapidly and applied in OLEDs taking design strategies like constructing D–A structure or embedding B units into highly luminescent frameworks (Wakamiya et al., 2007; Hoven et al., 2010). For solving the stability issue towards air and moisture of triarylboranes causing by B’s electrophilicity, bulky groups like two 2,4,6-mesityl (Mes) groups have been introduced to guarantee the stability via improving the steric hindrance around B, which is proved to be effective (Stahl et al., 2006; Zhao et al., 2009). The two bulky Mes group are twisted from the empty boron *p*_z_ orbital, and as a consequence, spin-orbital coupling are strongly enhanced, leading to triplet excited states and intense phosphorescence even at room temperature (Ji et al., 2017; Wu et al., 2020).

Carbazole (Cz) is an electron donor which have shown high luminescence performance and have been applied in OLED devices (Lin et al., 2008). It is easy to access their triplet excited state, which makes carbazole-containing compounds showing room-temperature persistent phosphorescent properties (Wang et al., 2011; Hudson et al., 2012). Carbazole derivatives have also shown distinguished properties of temperature-activated delayed-fluorescence (TADF) (Kitamoto et al., 2016; Liu et al., 2016; Higginbotham et al., 2018).

Compounds with Cz as electron donor and BMes_2_ as electron acceptor have been reported. While the 3,6-*bis*(BMes_2_)carbazole has shown strong fluorescence quantum yield and large two-photon absorption cross-sections (Cao et al., 2008), the 9-BMes_2_carbazole derivatives have exhibited interesting TADF properties shown high internal quantum efficiencies up to 100% in OLED devices (Lee et al., 2017; Lee et al., 2018).

Here we report two new carbazole derivatives, with BMes_2_ or 4-BMes_2_-phenyleneacetyl at 2,7-position of Cz. The additional introduction of aromatic phenylacetylene has both geometric and electronic effects on photophysical properties. In this paper, we report photoluminescence related properties of these compounds upon environmental change from fluid solution to solid solution and to solid powder, to explore their photophysical prospect in OLED materials.

## Materials and Methods

### Materials and Instruments

All reagents were commercially available and used as supplied without further purification. Ethyl ether, triethylamine and dioxane are re-steamed according to the solvent manual. ^1^H NMR and ^13^C NMR spectra were measured in CDCl_3_ at ambient temperature using a Bruker Advance 500 NMR spectrometer (operating at 500 MHz for ^1^H and 126 MHz for ^13^C). High resolution mass spectrometry (HRMS) was obtained by Thermo Fisher Scientific LTQ FTICR-MS and Thermo Scientific Q Exactive HF Orbitrap-FTMS. All photophysical measurements were performed in standard quartz cuvettes (1 cm × 1 cm cross-section). Luminogen-doped PMMA (polymethyl methacrylate) film was prepared by dissolving 2 mg of a luminogen compound and 100 mg of PMMA in 5 ml of specpure DCM followed by casting the resulting solution onto the surface of a quartz cell. UV-visible absorption spectra were recorded on a HITACHI UH5700 UV-visible spectrophotometer. The fluorescence, phosphorescence spectra, lifetimes and fluorescence quantum yields were recorded on a spectrofluorometer Edinburgh FLS1000. Thermogravimetric data were recorded on TGA5500.

### Synthesis

Compounds (Mes)_2_BF and (*p*-Iodophenyl)dimesitylborane were synthesized according to literature (Yuan et al., 2006) (Mes)_2_BF was obtained as white solid with 50% yield, ^1^HNMR (500 MHz, CDCl_3_) *δ* 6.86 (s, 4 H), 2.33 (s, 6 H), 2.30 (s, 12 H) (*p*-Iodophenyl)dimesitylborane was obtained as solid with 50% yield,^1^HNMR (500 MHz, CDCl_3_) *δ* = 7.69 (d, *J* = 8.1 Hz, 2 H), 7.20 (d, *J* = 8.1 Hz, 2 H), 6.81 (s, 4 H), 2.29 (s, 6 H), 1.98 (s, 12 H).

### 2,7-bis(dimesitylboraneyl)-9-ethyl-carbazole (BCz)

2,7-dibromo-9-ethyl-carbazole (0.565 g, 1.6 mmol) was placed in a 500 ml three-necked flask and was dried under vacuum and then backfilled with nitrogen before dry diethyl ether (20 ml) was added. The suspension was cooled to –78°C with vigorous stirring, and then n-BuLi in hexane (1.6 M, 2 ml, 3.2 mmol) was added. The suspension was stirred for further 2 h at –78°C. Mes_2_BF (0.858 g, 3.2 mmol) was then added and the mixture was stirred for another 2 h at –78°C before warming to room temperature and stirring overnight. Added a few drops of ethanol to quench the reaction. The obtained solid was dissolved in 200 ml dichloromethane and washed with water for three times. Isolated the organic layer. After the solvent was removed under vacuum, the residual was suspended in 100 ml ethanol, refluxed for 10 min, then filtered and rinsed with hot ethanol giving BCz as greenish solid powder (0.58 g, 52%). ^1^HNMR (500 MHz, CDCl_3_) *δ* = 8.09 (d, *J* = 7.8 Hz, 2 H), 7.61 (s, 2 H), 7.42 (d, *J* = 7.8 Hz, 2 H), 6.86 (s, 8 H), 4.26 (d, *J* = 7.1 Hz, 2 H), 2.34 (s, 12 H), 2.03 (s, 24 H), 1.26 (t, *J* = 7.0 Hz, 3 H); ^13^C{^1^H}NMR (126 MHz, CDCl_3_) *d* = 144.4, 142.2, 141.0, 140.8, 138.5, 128.2, 127.2, 125.6, 120.4, 116.8, 37.3, 23.6, 21.3, 14.1. HRMS (DART, POSITIVE): Calcd for C_50_H_55_B_2_N *m/z* = 692.4583; Found 692.4593.

### 9-ethyl-2,7-diethynyl-carbazole (ECz)

In an argon filled glove box, 2,7-dibromo-9-ethyl-carbazole (0.485 g, 1.37 mmol), PdCl_2_dppf (80 mg) and CuI (10 mg) were dissolved in dry paraxylene (10 ml) and triethylamine (10 ml) in a Schlenk tube. Then was heated to 100°C and stirred overnight. Reaction mixture was concentrated at reduced pressure, and crude products were purified by chromatography over silica (dichloromethane: petroleum, 1 : 1, v/v as the eluant) to obtain the title compound of white solid (0.272 g, 81%). ^1^HNMR (500 MHz, CDCl_3_) *δ* = 8.00 (d, *J* = 8.0 Hz, 2 H), 7.56 (s, 2 H), 7.37 (d, *J* = 8.0 Hz, 2 H), 4.33 (q, *J* = 7.2 Hz, 2 H), 3.16 (s, 2 H), 1.43 (t, *J* = 7.2 Hz, 3 H); ^13^C{^1^H} NMR (126 MHz, CDCl_3_) *δ* = 140.1, 123.3, 123.0, 120.6, 119.4, 112.6, 84.8, 76.9, 37.7, 13.8.

### 2,7-bis((4-(dimesitylboraneyl)phenyl)ethynyl)-9-ethyl-carbazole (BPACz)

In an argon filled glove box, 9-ethyl-2,7-diethynyl-carbazole (48.6 mg, 0.2 mmol) (*p*-Iodophenyl)dimesitylborane (180.9 mg, 0.4 mmol), CuI (4 mg) and Pd(PPh_3_)_4_ (7 mg) were dissolved in dry dioxane (10 ml) and triethylamine (1.5 ml) in a Schlenk tube. Stirred overnight at room temperature. Reaction mixture concentrated at reduced pressure, crude products were purified by silica-gel column chromatography using mixture solvent (chloroform: petroleum, 1: 4, v/v as the eluant) to obtain a white solid (150 mg, 84%). ^1^HNMR (500 MHz, CDCl_3_) *δ* = 8.05 (d, *J* = 8.1 Hz, 2 H), 7.63 (s, 2 H), 7.61–7.53 (m, 8 H), 7.45 (d, *J* = 8.0 Hz, 2 H), 6.86 (s, 8 H), 4.39 (d, *J* = 7.2 Hz, 2 H), 2.34 (s, 12 H), 2.05 (s, 24 H), 1.48 (t, *J* = 7.2 Hz, 3 H); ^13^C{^1^H} NMR (126 MHz, CDCl_3_) *δ* = 141.6, 140.9, 140.3, 138.9, 136.2, 131.1, 128.3, 126.8, 123.1, 122.9, 120.6, 120.4, 112.0, 93.0, 89.8, 37.8, 23.5, 21.3, 13.9. HRMS(AP-MALDI Positive Ion Mode): Calcd for C_66_H_63_B_2_N *m/z* = 891.5135; Found 891.5141.

### Measurement

NMR spectra were recorded in CDCl_3_, HRMS were performed in DART Positive and AP-MALDI Positive ionization Mode on a u-TOF mass spectrometer. All absorption and emission spectra were collected at *c* = 1.0 × 10^−6^ mol/L. The absorption spectra, fluorescence emission spectra, and fluorescence lifetime (*τ*_*F*_) were collected at room temperature, and the phosphorescence emission spectra and phosphorescence lifetime were collected at 77 K. The fluorescence quantum yields (*Φ*_*F*_) for the compounds were determined relative to anthracene using a standard method.

### Theoretical Calculations

DFT (density functional theory) calculations of two compounds were performed by using Gaussian 16 (Frisch et al., 2016) package. The geometries of ground state (S_0_) of BCz and BPACz were optimized under Cs and C1 symmetry restriction in vacuum, respectively. The functional used includes Becke’s three parameter hybrid functional (Becke, 1993) in conjunction with the Lee–Yang–Parr correlation functional (Lee et al., 1988), which is abbreviated as B3LYP. The basis set 6-311G* was employed. Molecular orbitals were visualized with Gauss View 6.0 (Inc, G., 2006) and the total volume of a molecule was estimated by Multiwfn (Lu et al., 2012) package from DFT optimized geometries.

## Results and Discussion

### Absorption and Emission (PL) Properties

We examined the absorption and emission properties of both compounds in three different matrices, from flowing molecule-dispersed solution to the condensed, aggregated state. PMMA is introduced as a transition medium which can exert rigid environment on dispersed molecule, i.e., solid solution.

**FIGURE 1:**
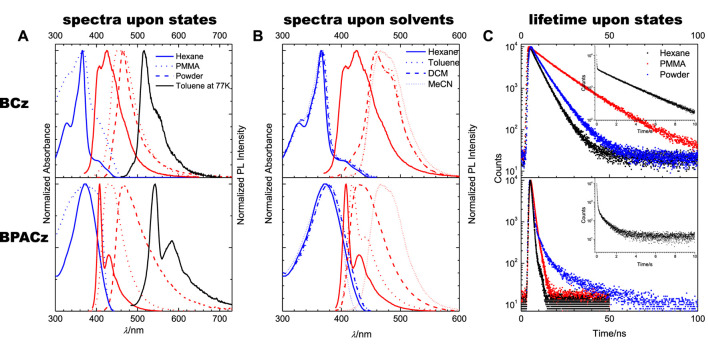
Absorption and PL properties of BCz (top) the BPACz (bottom): **(A)** spectra (Absorption in blue, PL in red) in solution of hexane (solid lines), PMMA (dot lines) and powder (dashed lines) at room temperature and PL spectra in toluene at 77 K (in black solid line). **(B)** spectra (Absorption in blue, PL in red) in different solvents (hexane, solid lines; toluene, dot lines; DCM, dashed lines; MeCN, short dot lines) at room temperature. **(C)** PL lifetimes of BCz and BPACz in hexane and PMMA, excited at 380 nm, the one of powder excited at 295 nm; the insets are PL lifetimes in toluene at 77 K.

Two aspects of the properties are evaluated, one is the change of the spectral aspect (position and bandshape) and the other is the evolution of photophysics (PL quantum yield and lifetime). The results of both compounds in variable environment will be logically discussed in a systematically comparative way: from unbridged BCz to BPACz, from liquid solution to PMMA and to powder. The UV-Vis Absorption and PL spectra of two compounds are plotted in Figure 1, see the corresponding maxima in Table 1; PL quantum yields (*Φ*_*F*_) and lifetimes (*τ*_*F*_) are shown as well.

**SCHEME 1:**
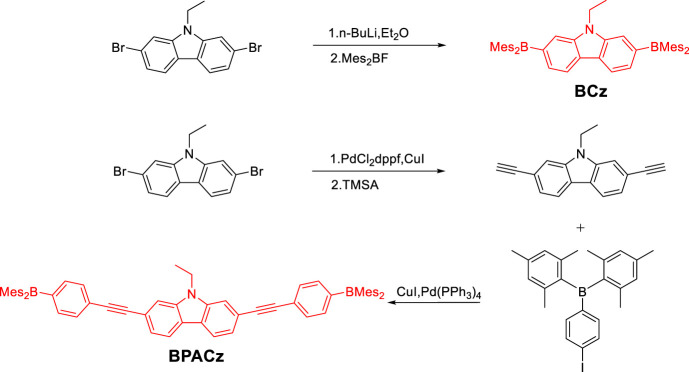
Synthetic strategy of the title compounds.

**TABLE 1:** Photophysical properties of BCz and BPACz in different status: four solutions (hexane, toluene, DCM, MeCN); PMMA (polymethylmethacrylate) film; pristine powder. Maximum of absorption and emission ( $\lambda_{abs}$ , $\lambda_{em}$ ), PL quantum yield and lifetime (*Φ*_F_,*τ*_F_), stokes shift $\Delta \bar{\nu }$ .

Status	$\lambda_{abs}^{\max}/nm$ ^a^		$\lambda_{em}^{\max}/nm$ ^a^		${\Delta \bar{\nu }/cm}^{-1}$ ^b^		*Φ*_*F*_ ^c^		*τ*_*F*_/ns^d^	
Comp	BCz	BPACz	BCz	BPACz	BCz	BPACz	BCz	BPACz	BCz	BPACz
Hexane	328,365,407	372	407,425	408,430	3,867	2,371	0.49	0.95	3.9(m)	0.6(b)
Toluene	333,369,403	379	458,479	429	5,266	3,075	—	—	11.8(m)	0.7(b)
DCM	332,367,403	375	458,482	419,442	5,413	2,800	—	—	—	—
CH_3_CN	331,364,401	370	466	458	6,013	5,192	—	—	—	—
PMMA	—	—	458	431	—	—	0.60	0.92	13.2(m)	1.2(b)
Powder	—	—	465	466	—	—	0.13	0.16	6.1(m)	0.9(t)

a All the subbands are listed if there is, and the strongest peak is underlined.

b Stokes shift is the difference between the maximum (in wave number) of absorption and PL emission.

c Fluorescence quantum yields determined using anthracene (in ethanol) as standard.

d Lifetimes measured in hexane, PMMA and powder upon excitation of 380 nm, 380 and 295 nm, respectively. Monoexponential, biexponential and triexponential fit are denoted as m, b, t in parentheses, respectively.

For BCz in hexane, the PL spectrum is vibronically structured with two subbands positioned at 400 and 430 nm, which were assigned as apparent 0–0 and 0–1 bands. Compared to the PL spectrum, the absorption spectrum is also structured; a maximum peak at 340 nm and a weak shoulder peak at 406 nm are observed. With respect to the photophysical parameters, BCz is showing strong emission with a quantum yield of *Φ*_*F*_ = 0.49 and a corresponding lifetime of *τ*_*F*_ = 3.94 ns. Compared to 3,6-BCz reported earlier, 2,7-BCz in hexane gives slightly bule-shifted absorption but red-shifted fluorescence with similar quantum yield (Cao et al., 2008).

Compared to BCz in hexane, BPACz with PA bridge showed non-shifted PL with more pronounced vibronic feature and slightly red-shifted absorption (maximum peak at 372 nm). It also shows a much higher quantum yield (*Φ*_*F*_ = 0.95) with a much shorter lifetime (*τ*_*F*_ = 0.6 ns), which is probably because of the fast radiative decay rate (1.6 × 10^9^ s^−1^) compared to BCz (1.3 × 10^8^ s^−1^), while the non-radiative decay rates of the two compounds keep similar.

When going from hexane to more polarized solvents (toluene, DCM, MeCN), we observed little dependence in absorption while a general bathochromic shift and substructure washing out in PL for both compounds. The bathochromic shift implies that the polarity in excited state is much larger than that in ground state. The difference in dipole moment Δ*μ*_ge_ between excited state and ground state can be estimated by Lippert–Mataga equation (Lakowicz, 1983):

$$\begin{array}{l} \Delta \bar{v}={\bar{v}}_{abs}-{\bar{v}}_{em}=2\Delta \mu_{ge}^{2}\Delta f/hca^{3}+const,\\ \Delta f=\left[\left(\varepsilon -1\right)/\left(2\varepsilon +1\right)\right]\left[\left(n^{2}-1\right)/\left(2n^{2}+1\right)\right] \end{array}$$ (1)

where *h* is Planck’s constant, *c* is the speed of light, *a* is the cavity radius of the molecule, Δf is the orientation polarizability reflecting the polarity of solvent and can be obtained via equation above where *ε* is the dielectric constant and *n* is the refractive index of solvent. We plotted stokes shifts $\left(\Delta \bar{v}\right)$ against the orientational polarizability (Δf) (see Supplementary Figure S1), which is basically proportional with the slope of 4.76·10^3^ and 6.33·10^3^ cm^−1^ for the fitted lines of BCz and BPACz, respectively. By assuming the molecule to be a sphere, *a* is equivalent to 6.1 Å for BCz and 6.6 Å for BPACz, obtained from the total volume of a molecule given by the theory geometry optimization procedure. With the slope and other values known, Δ*μ*_ge_ is obtained as 10.3 D for BCz and 13.4 D for BPACz.

The phosphorescence of BCz and BPACz in frozen solutions were also investigated, and the results are listed in Figure 1A. When cooling the temperature to 77 K, the toluene solutions of both compounds show strong phosphorescence around 516 nm for BCz and 540 nm for BPACz with lifetime of 2.9 and 0.4 s, respectively. As we can see, the phosphorescence spectra are vibronically structured as fluorescence, which suggests that the vibronic coupling modes at room temperature are still active at low temperature.

When going from fluid solution (hexane) to solid solution (PMMA), the absorption maxima are similar to that in fluid solution, however spectral shape gets blurred; the PL spectra for both compounds show significant redshift and less vibronic features in PMMA against hexane, similar to the case in polarized solvents. The significant redshift of PL indicates that excited state is of much larger polarity than ground state as we concluded from solvent dependency above. Giving that the rigidity of solid matrix should have a significant effect on narrowing the shape of the torsional potential along with giving more pronounced vibronic structure in the corresponding spectrum, the blurring of spectral shape in PMMA against hexane above demonstrates that PMMA can hardly steepen the torsional potentials in S0 and S1 because of free volume existing in PMMA making it different from the really rigid and highly ordered environments such as perhydrotriphenylene (Shi et al., 2017a; Shi et al., 2017b), implying that the torsional potentials of ground and excited states are quite steep.

As seen in Figure 1C, both compounds show (substantially) increased lifetimes when going from dilute solution to the PMMA matrix, which is related to the largely limited nonradiative decay pathways in the relatively rigid environment provide by PMMA. PMMA films show similar quantum yields to that in solutions for both compounds (see Table 1), which implies that PMMA also has a reduction effect on radiative decay probably due to its polarizability.

Besides the non-aggregated solid state (i.e., PMMA), the “classical” solid state was investigated, where intermolecular interactions can also significantly influence the emission property. In our case, both compounds show red-shifted and blurred PL in powder against hexane, as well as increased lifetimes to different extent. The former is due to the polarizability of the rigid matrix while the latter is resulted from the suppressed nonradiative rates by the rigidity exerting on the molecular motion. Amorphous powder samples of two compounds show decreased quantum yields compared to solutions (see Table 1), which suggests that current amorphous environment affects the radiative part significantly probably due to strong exciton trap by high surface-to-volume ratio in amorphous state. We also performed TGA test and the results see Supplementary Figure S10–11 in SI. BCz shows higher than 260°C for 5% loss while BPACz shows 160°C.

### DFT Calculations

**FIGURE 2:**
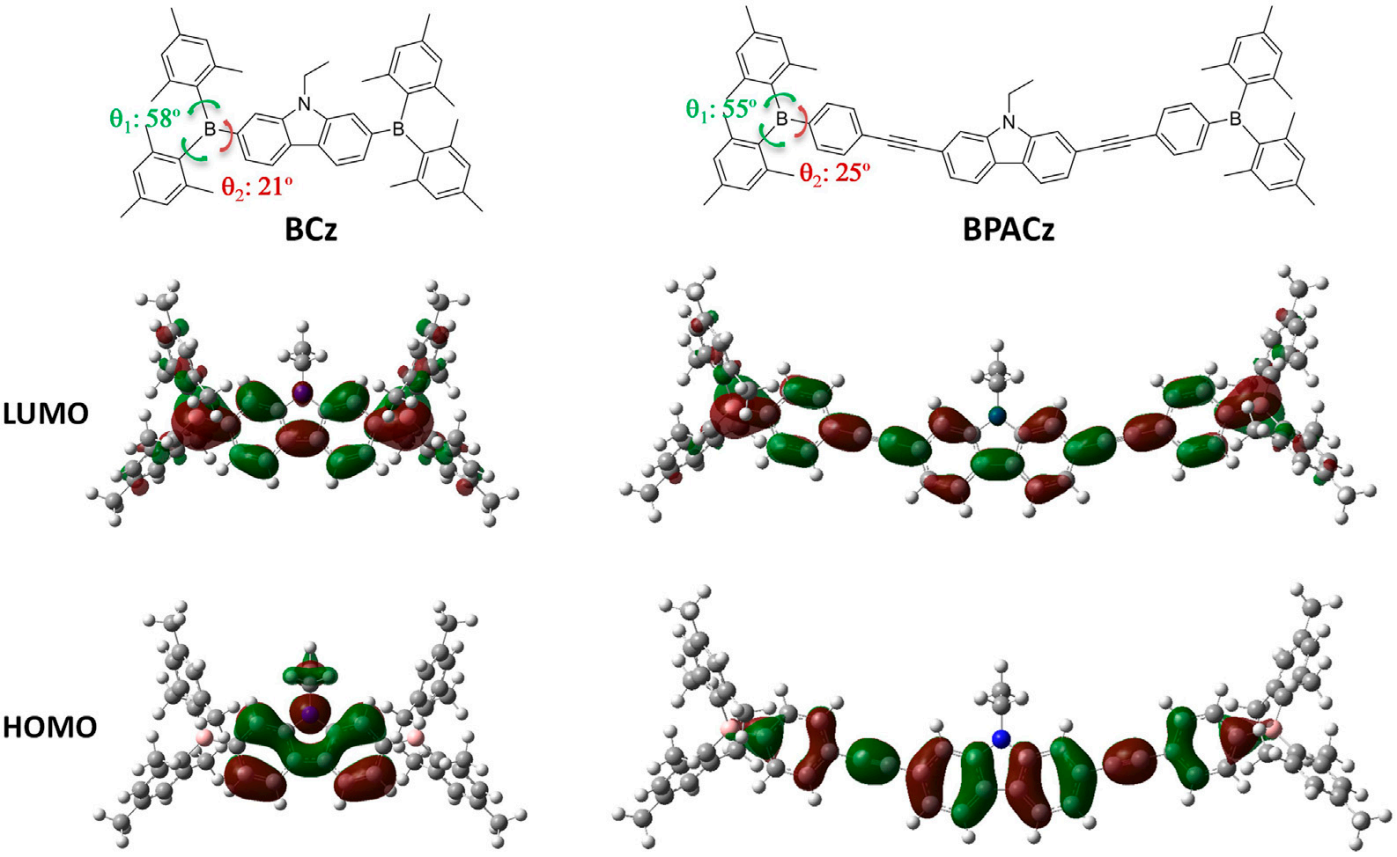
The electron density distribution of the frontier molecular orbitals of the compounds under study.

The equilibrium geometries of both compounds are elucidated by DFT (in vacuum) via full optimization under specific symmetry restrictions (Cs for BCz, C1 for BPACz). Relevant torsional angles are denoted in Figure 2 and the corresponding values are listed beside.

The results of torsional angles suggest that strong twists in the structure is a general signature for the BMes_2_-containing compounds, which however vary upon substitution (pattern, position, orientation) and in particular sharply upon the environment change such as from solution to single crystal; in this paper only the torsion in vacuum are described, the one in single crystal are not done because single crystals are hard to obtain probably due to the poor crystallinity of these compounds. It’s known from earlier studies that Cz exhibits a planar equilibrium geometry (Brown and Dodson, 1957) while BMes_2_ are twisted due to the steric hindrance between the neighboring benzenes. Therefore, BCz exhibits a nonplanar equilibrium geometry with a planar core (Cz) and torsional ends (BMes_2_); the introduction of PA groups retains this end-twisted geometry in BPACz and only expands the planar core with electronic effect but without geometrical effect. From the experimental results that BCz has similar spectral position to BPACz, we suggest PA’s electronic effect is quite trivial. From the fact that two compounds show little spectral shift upon environment changes, we assume that the torsional potentials around the B-C single bonds are not shallow enough for environmental restriction to exert influence.

We also investigated the MO’s topologies of two compounds with the electron density distribution of the frontier molecular orbitals (HOMO and LUMO) illustrated in Figure 2. For BCz, the electrons are mainly concentrated on the N atom and Cz ring in the HOMO while the electrons are spreading to the boron atoms in the LUMO, which indicates that charge transfer from N-ethyl-carbazole to boron atoms upon excitation from HOMO to LUMO. For BPACz, the trend of electrons spreading from central part to BMes_2_ end is also the case but with less intensity, which explains the appearance of the strong charge transfer band around 407 nm observed in the absorption spectrum of BCz.

## Conclusion

Two new A(B)-D (Cz)-A(B) type organoboron compounds, BCz and BPACz have been synthesized and their photoluminescence property has been investigated. Structured PL around 410 nm with high quantum yield and less structured absorption are found for BCz in hexane, which is more pronounced for BPACz. Both compounds in toluene are phosphorescent at 77 K with long lifetime up to 3 s for BPACz. Absorption spectra show little dependence on solvent polarity while PL shows a general redshift and substructure blurring because the polarity in excited state is much larger than that in ground state. Red-shifted and blurred PL in powder/PMMA against hexane, as well as increased lifetimes to different extent are found; the former is due to the polarizability of the matrix affecting on more polarized excited states while the latter is related to the suppressed nonradiative rates by the matrix’s rigidity exerting on the molecular motion. Quantum yields of PMMA are similar to that of solutions while show a decrease in amorphous powder, which is a reflection of rigid environment’s influence on radiative part. From DFT calculation, we know both molecules exhibit an end-twisted geometry and a tendency of charge transfer from N-ethyl-carbazole to boron atoms upon excitation from HOMO to LUMO. In sum, these two compounds of BMes_2_-Cz structure are potential blue emitting materials which can be exploited further in optoelectronic application.

## Data Availability

The original contributions presented in the study are included in the article/Supplementary Material, further inquiries can be directed to the corresponding author.
