# Supplementary material for: 2,7-Carbazole Derived Organoboron Compounds: Synthesis and Molecular Fluorescence
Source: Front Chem. 2021 Oct 22;9:754298. doi: 10.3389/fchem.2021.754298 (PMC8568956; doi:10.3389/fchem.2021.754298)
Supplement: Supplementary file 1 [file DataSheet1.docx]

**2,7- Carbazole Derived Organoboron Compounds: Synthesis and Molecular Fluorescence**

Minhui Chen, Juan Wei, Yufeng Zhang, Lin Wu, Leibo Tan, Shanglong Shi, Junqing Shi,^*^ Lei Ji

Frontiers Science Center for Flexible Electronics, Xi’an Institute of Flexible Electronics (IFE) and Xi’an Institute of Biomedical Materials & Engineering, Northwestern Polytechnical University, 127 West Youyi Road, Xi'an 710072, China

^*^ email: [shi.junqing@nwpu.edu.cn](mailto:shi.junqing@nwpu.edu.cn)

**Supplementary Figures and Tables**

**Figure S1** ^1^H NMR spectrum (500 MHz) of **BCz**.

**Figure S2** ^13^C NMR spectrum (126 MHz) of **BCz**.

**Figure S3** DART POSITIVE Mode HRMS of **BCz**.

**Figure S4** ^1^H NMR spectrum (500 MHz) of **BPACz**.

**Figure S5** ^13^C NMR spectrum (126 MHz) of **BPACz**.

**Figure S6** AP-MALDI Positive Ion Mode HRMS of **BPACz**.

**Figure S6** AP-MALDI Positive Ion Mode HRMS of **BPACz**.

**Figure S7** ^1^H NMR spectrum (500 MHz) of **ECz**.

**Figure S8** ^13^C NMR spectrum (126 MHz) of **ECz**.

**Figure S9** Stokes shift $\Delta\bar{\nu}$ vs orientational polarizability $\Delta f$of the solvents.

**Figure S10** Thermogravimetric data of **BCz.**

**Figure S11** Thermogravimetric data of **BPACz.**

**Table S1** XZY coordinates of compound **BCz**.

**Table S2** XZY coordinates of compound **BPACz**.


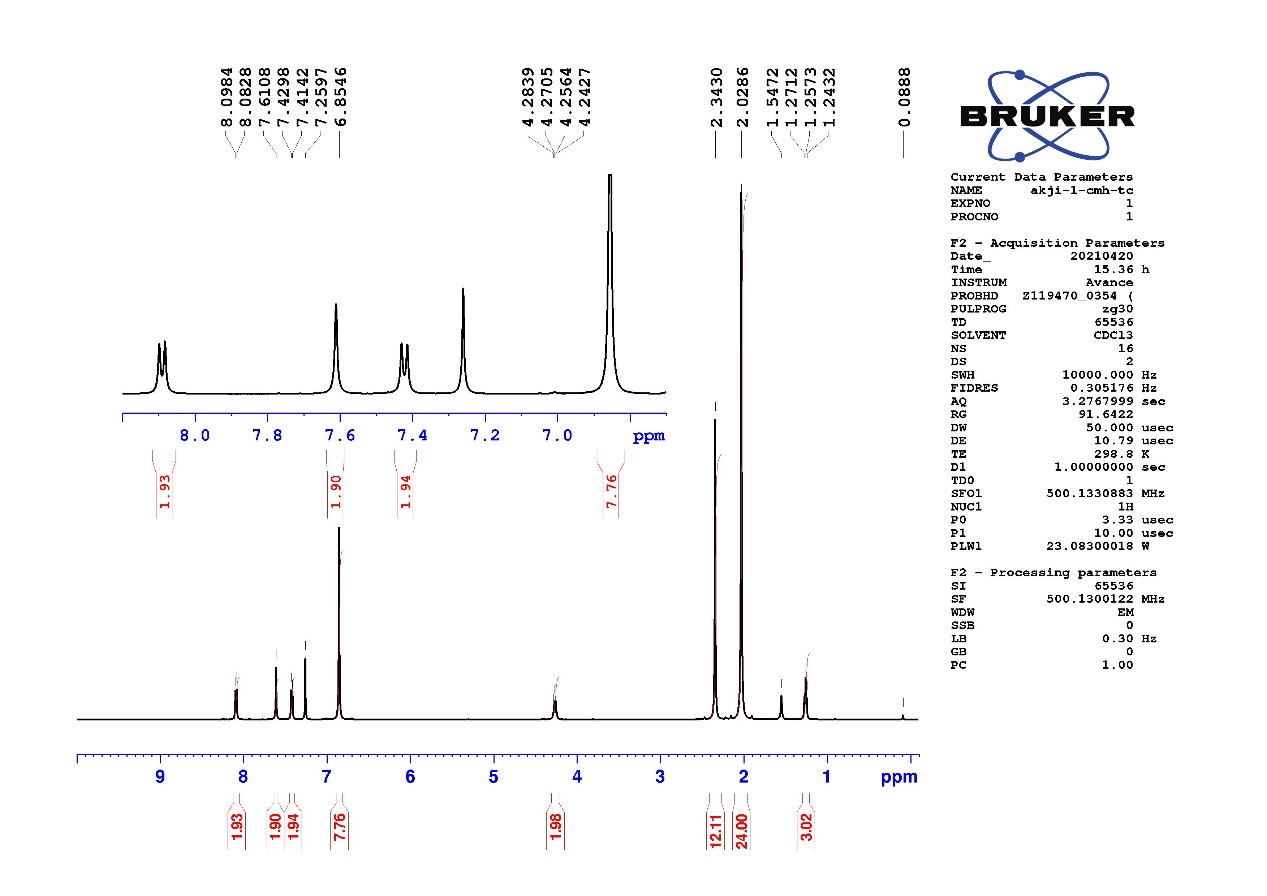


**Figure S1** ^1^H NMR spectrum (500 MHz) of **BCz** in CDCl_3_ solution(298K).


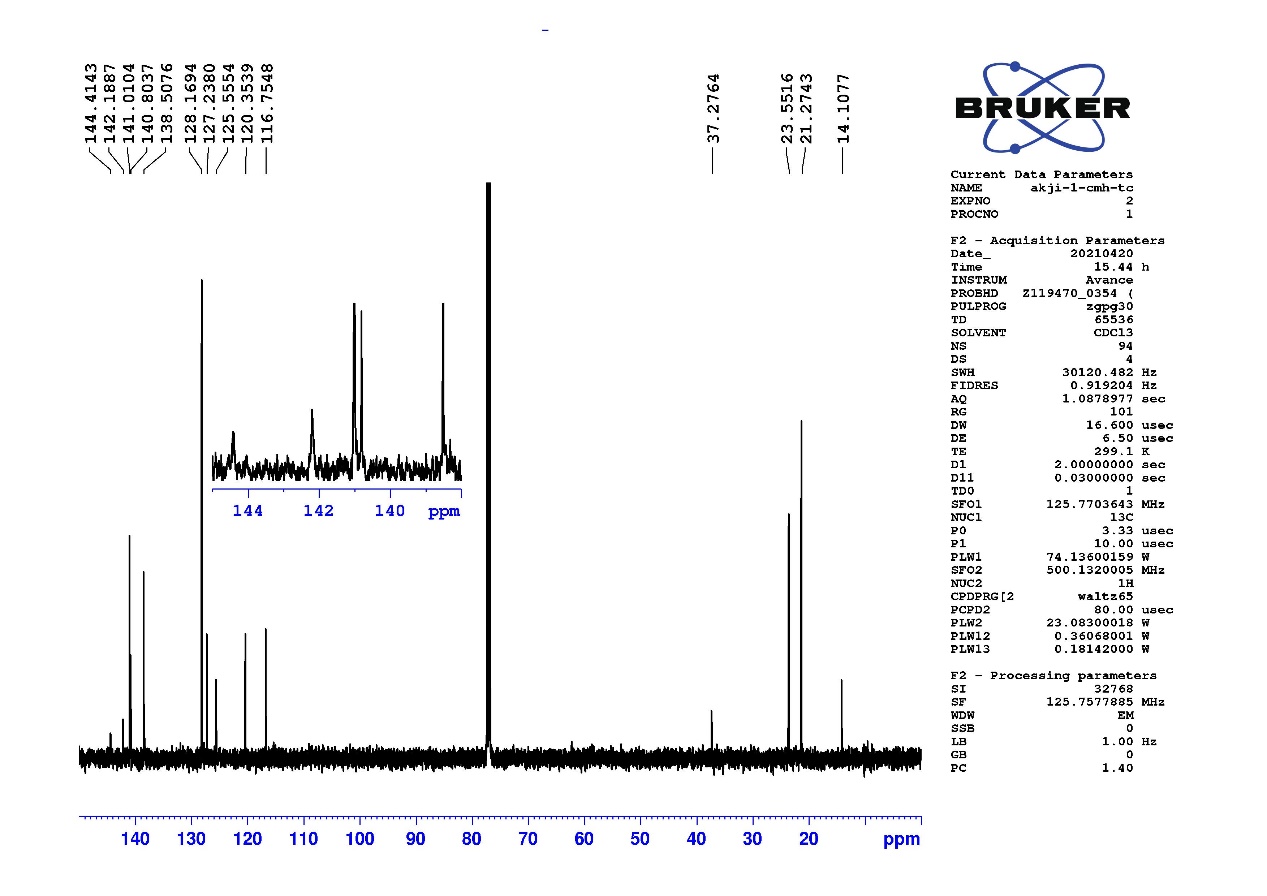


**Figure S2** ^13^C NMR spectrum (126 MHz) of **BCz** in CDCl_3_ solution(298K).

**Figure S3** DART POSITIVE Mode HRMS of **BCz**.


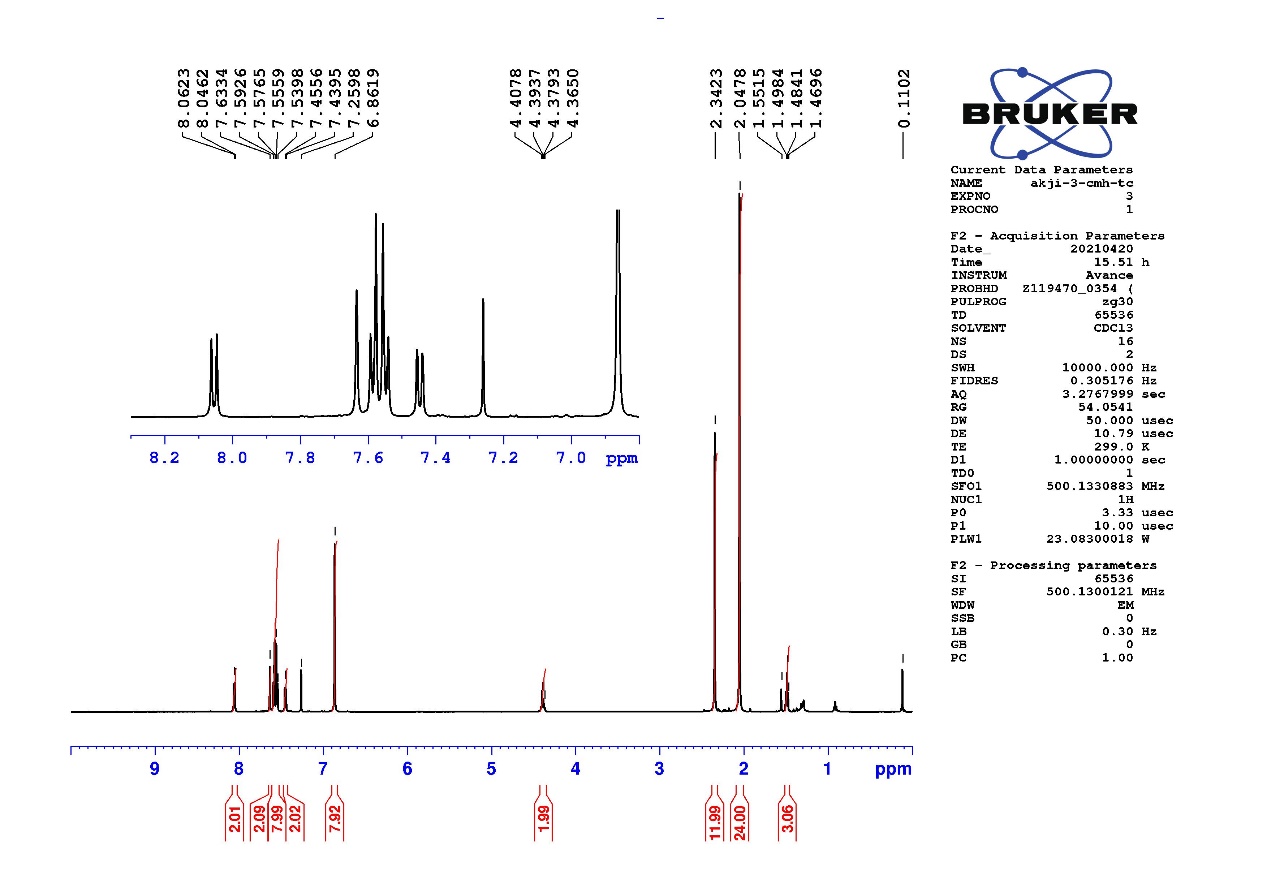


**Figure S4** ^1^H NMR spectrum (500 MHz) of **BPACz** in CDCl_3_ solution(298K).


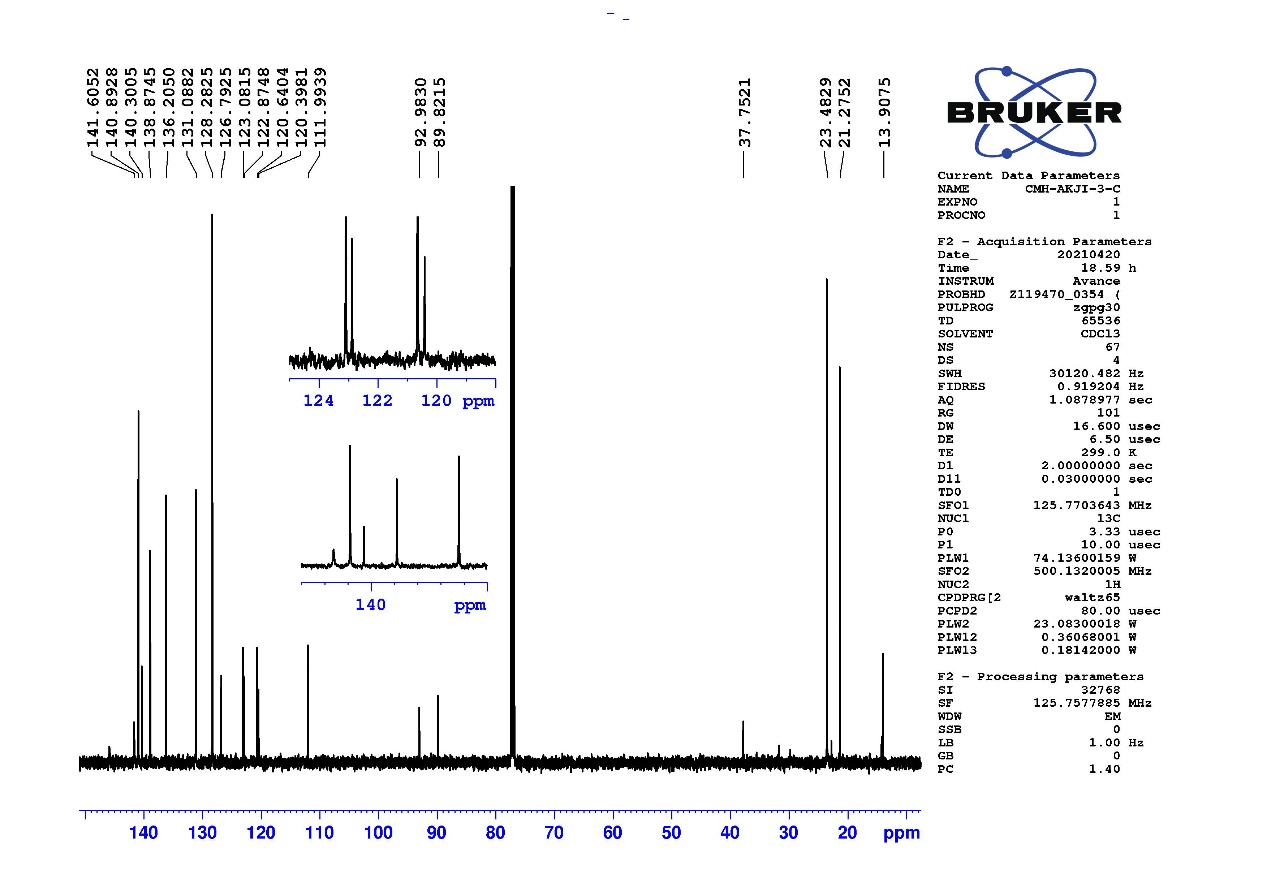


**Figure S5** ^13^C NMR spectrum (126 MHz) of **BPACz** in CDCl_3_ solution(298K).

**Figure S6** AP-MALDI Positive Ion Mode HRMS of **BPACz**.


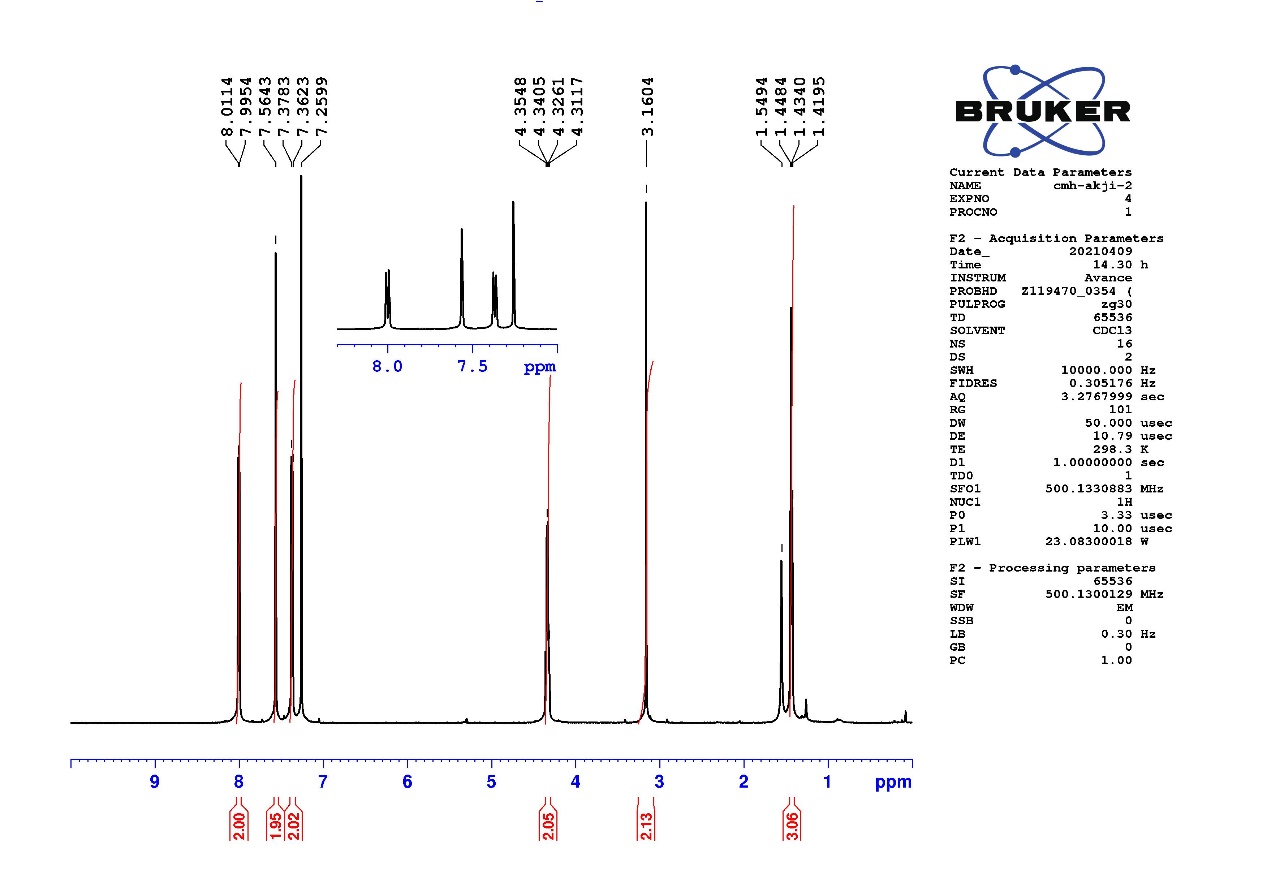


**Figure S7** ^1^H NMR spectrum (500 MHz) of **ECz** in CDCl_3_ solution(298K).


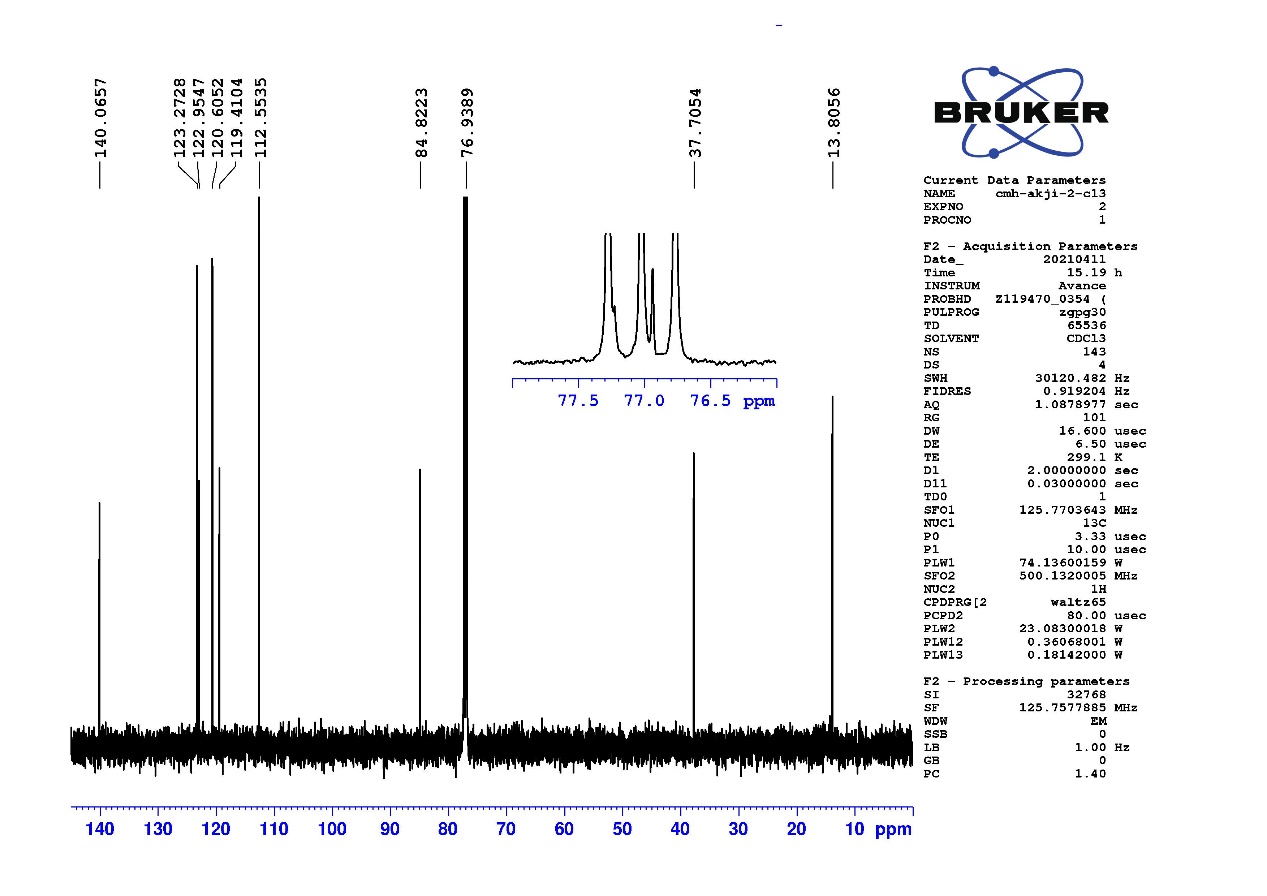


**Figure S8** ^13^C NMR spectrum (126 MHz) of **ECz** in CDCl_3_ solution(298K).

**Figure S9** Stokes shift $\Delta\bar{\nu}$ vs orientational polarizability $\Delta f$of the solvents: the scatter dots are the experimental data and the line is the linearly fitted result.

 **Figure S10** Thermogravimetric data of **BCz.**

**Figure S11** Thermogravimetric data of **BPACz.**

**Table S1** XZY coordinates of compound **BCz**.

---------------------------------------------------------------------

Center Atomic Atomic Coordinates (Angstroms)

Number Number Type X Y Z

---------------------------------------------------------------------

1 6 0 1.821341 0.772650 -3.032436

2 6 0 0.558401 0.277381 -3.455348

3 6 0 -0.382788 -0.085079 -2.475294

4 6 0 -0.052796 0.039033 -1.127639

5 6 0 1.211281 0.545222 -0.720169

6 6 0 2.145439 0.919645 -1.693900

7 1 0 2.549586 1.049367 -3.786755

8 1 0 -1.352364 -0.462464 -2.780882

9 1 0 3.114452 1.314004 -1.403034

10 6 0 -0.052796 0.039033 1.127639

11 6 0 -0.382788 -0.085079 2.475294

12 6 0 0.558401 0.277381 3.455348

13 6 0 1.821341 0.772650 3.032436

14 6 0 2.145439 0.919645 1.693900

15 6 0 1.211281 0.545222 0.720169

16 1 0 -1.352364 -0.462464 2.780882

17 1 0 2.549586 1.049367 3.786755

18 1 0 3.114452 1.314004 1.403034

19 7 0 -0.810125 -0.253982 0.000000

20 5 0 0.214006 0.131478 -4.978827

21 6 0 -1.309582 0.063561 -5.414270

22 6 0 -1.811856 -1.075037 -6.104284

23 6 0 -2.221856 1.099172 -5.104609

24 6 0 -3.162507 -1.155561 -6.433523

25 6 0 -3.569088 0.986440 -5.471083

26 6 0 -4.064314 -0.131621 -6.130135

27 1 0 -3.526181 -2.044130 -6.944857

28 1 0 -4.245277 1.804762 -5.232071

29 6 0 1.391791 0.047994 -6.035616

30 6 0 1.483284 0.994887 -7.094350

31 6 0 2.397966 -0.942505 -5.952498

32 6 0 2.545848 0.941965 -7.992252

33 6 0 3.438244 -0.972844 -6.889747

34 6 0 3.538258 -0.039176 -7.913044

35 1 0 2.603741 1.687919 -8.781802

36 1 0 4.189959 -1.755451 -6.809638

37 5 0 0.214006 0.131478 4.978827

38 6 0 -1.309582 0.063561 5.414270

39 6 0 -2.221856 1.099172 5.104609

40 6 0 -1.811856 -1.075037 6.104284

41 6 0 -3.569088 0.986440 5.471083

42 6 0 -3.162507 -1.155561 6.433523

43 6 0 -4.064314 -0.131621 6.130135

44 1 0 -4.245277 1.804762 5.232071

45 1 0 -3.526181 -2.044130 6.944857

46 6 0 1.391791 0.047994 6.035616

47 6 0 1.483284 0.994887 7.094350

48 6 0 2.397966 -0.942505 5.952498

49 6 0 2.545848 0.941965 7.992252

50 6 0 3.438244 -0.972844 6.889747

51 6 0 3.538258 -0.039176 7.913044

52 1 0 2.603741 1.687919 8.781802

53 1 0 4.189959 -1.755451 6.809638

54 6 0 -2.148103 -0.826921 0.000000

55 1 0 -2.678224 -0.441020 -0.873990

56 1 0 -2.678224 -0.441020 0.873990

57 6 0 -2.157118 -2.357476 0.000000

58 1 0 -1.652157 -2.753545 -0.883924

59 1 0 -1.652157 -2.753545 0.883924

60 1 0 -3.184726 -2.731677 0.000000

61 6 0 2.392322 -2.051348 -4.918618

62 1 0 2.288964 -3.027406 -5.404924

63 1 0 3.333555 -2.072196 -4.360853

64 1 0 1.589588 -1.959713 -4.190335

65 6 0 0.466999 2.104257 -7.265002

66 1 0 0.406782 2.745014 -6.380528

67 1 0 0.730653 2.744618 -8.109413

68 1 0 -0.536699 1.712766 -7.443912

69 6 0 4.672330 -0.071091 -8.907748

70 1 0 4.301362 -0.141961 -9.935154

71 1 0 5.279001 0.838592 -8.850082

72 1 0 5.335202 -0.921618 -8.734906

73 6 0 -0.921138 -2.242890 -6.472886

74 1 0 -0.118418 -1.943585 -7.150086

75 1 0 -0.445668 -2.690833 -5.595364

76 1 0 -1.497110 -3.029698 -6.964659

77 6 0 -1.807414 2.399471 -4.444933

78 1 0 -1.882685 3.229839 -5.155579

79 1 0 -2.465285 2.641877 -3.604858

80 1 0 -0.788152 2.384701 -4.064694

81 6 0 -5.518607 -0.244934 -6.516360

82 1 0 -6.094737 0.616266 -6.171186

83 1 0 -5.638794 -0.309435 -7.602743

84 1 0 -5.977002 -1.143660 -6.091588

85 6 0 -0.921138 -2.242890 6.472886

86 1 0 -0.118418 -1.943585 7.150086

87 1 0 -1.497110 -3.029698 6.964659

88 1 0 -0.445668 -2.690833 5.595364

89 6 0 -1.807414 2.399471 4.444933

90 1 0 -0.788152 2.384701 4.064694

91 1 0 -2.465285 2.641877 3.604858

92 1 0 -1.882685 3.229839 5.155579

93 6 0 -5.518607 -0.244934 6.516360

94 1 0 -5.638794 -0.309435 7.602743

95 1 0 -6.094737 0.616266 6.171186

96 1 0 -5.977002 -1.143660 6.091588

97 6 0 0.466999 2.104257 7.265002

98 1 0 0.406782 2.745014 6.380528

99 1 0 -0.536699 1.712766 7.443912

100 1 0 0.730653 2.744618 8.109413

101 6 0 2.392322 -2.051348 4.918618

102 1 0 2.288964 -3.027406 5.404924

103 1 0 1.589588 -1.959713 4.190335

104 1 0 3.333555 -2.072196 4.360853

105 6 0 4.672330 -0.071091 8.907748

106 1 0 5.279001 0.838592 8.850082

107 1 0 4.301362 -0.141961 9.935154

108 1 0 5.335202 -0.921618 8.734906

--------------------------------------------------------------------

**Table S2** XZY coordinates of compound **BPACz**.

---------------------------------------------------------------------

Center Atomic Atomic Coordinates (Angstroms)

Number Number Type X Y Z

---------------------------------------------------------------------

1 6 0 -3.032136 -2.929813 0.034026

2 6 0 -3.435299 -1.570662 0.059847

3 6 0 -2.470874 -0.549785 0.075867

4 6 0 -1.126888 -0.909420 0.068903

5 6 0 -0.719750 -2.270762 0.038584

6 6 0 -1.692659 -3.276602 0.023408

7 1 0 -3.795968 -3.698274 0.021082

8 1 0 -2.789518 0.485537 0.088543

9 1 0 -1.403537 -4.322476 0.002268

10 6 0 1.127855 -0.909312 0.057637

11 6 0 2.471806 -0.549554 0.051083

12 6 0 3.436137 -1.570360 0.026566

13 6 0 3.032879 -2.929563 0.005623

14 6 0 1.693387 -3.276462 0.007672

15 6 0 0.720572 -2.270699 0.031610

16 1 0 2.790466 0.485800 0.060299

17 1 0 3.796621 -3.697968 -0.014156

18 1 0 1.404169 -4.322370 -0.010239

19 7 0 0.000594 -0.098424 0.093050

20 6 0 0.000477 1.357026 0.083219

21 1 0 -0.869722 1.695415 0.650968

22 1 0 0.876261 1.695498 0.642264

23 6 0 -0.006580 1.959376 -1.323820

24 1 0 -0.006283 3.051631 -1.269773

25 1 0 -0.892695 1.647666 -1.881249

26 1 0 0.873885 1.647610 -1.890104

27 6 0 -4.817401 -1.242132 0.065032

28 6 0 -5.997752 -0.967767 0.068499

29 6 0 4.818165 -1.241593 0.018756

30 6 0 5.998384 -0.966726 0.011837

31 6 0 -7.381414 -0.649437 0.070138

32 6 0 -7.814167 0.689512 0.133999

33 6 0 -8.354292 -1.666185 0.006655

34 6 0 -9.167674 0.989456 0.149242

35 1 0 -7.074691 1.481812 0.180289

36 6 0 -9.703127 -1.346221 -0.010031

37 1 0 -8.033721 -2.701418 -0.039012

38 6 0 -10.155504 -0.013206 0.068675

39 1 0 -9.474807 2.028518 0.216307

40 1 0 -10.432164 -2.147705 -0.077614

41 6 0 7.381741 -0.647202 0.003191

42 6 0 7.812758 0.693844 0.001407

43 6 0 8.355982 -1.664573 -0.003987

44 6 0 9.165613 0.995976 -0.023692

45 1 0 7.072254 1.486485 0.010529

46 6 0 9.704801 -1.344093 0.003784

47 1 0 8.036704 -2.701210 -0.006209

48 6 0 10.155121 -0.008205 -0.014627

49 1 0 9.471058 2.037545 -0.042154

50 1 0 10.435306 -2.147005 0.015833

51 5 0 -11.684518 0.337380 0.065442

52 5 0 11.683775 0.343532 -0.024173

53 6 0 12.144557 1.726007 -0.642976

54 6 0 12.920221 2.647257 0.111121

55 6 0 11.791047 2.088242 -1.966459

56 6 0 13.291878 3.870456 -0.445925

57 6 0 12.210225 3.312329 -2.495996

58 6 0 12.951234 4.225490 -1.751802

59 1 0 13.874599 4.565195 0.154612

60 1 0 11.948873 3.555522 -3.523477

61 6 0 12.708955 -0.697578 0.585184

62 6 0 13.799713 -1.190463 -0.180724

63 6 0 12.563672 -1.175366 1.911192

64 6 0 14.672601 -2.129551 0.367619

65 6 0 13.479540 -2.094607 2.431517

66 6 0 14.535042 -2.595935 1.675709

67 1 0 15.492472 -2.502604 -0.241892

68 1 0 13.361636 -2.425963 3.460812

69 6 0 -12.158945 1.686994 0.743602

70 6 0 -12.919272 2.645630 0.020700

71 6 0 -11.832243 1.982506 2.090075

72 6 0 -13.301805 3.839878 0.630204

73 6 0 -12.261874 3.179387 2.671428

74 6 0 -12.987577 4.129088 1.958986

75 1 0 -13.871989 4.564446 0.053301

76 1 0 -12.020947 3.370921 3.714702

77 6 0 -12.695949 -0.672042 -0.616022

78 6 0 -13.800933 -1.202884 0.102360

79 6 0 -12.523418 -1.083408 -1.961023

80 6 0 -14.661077 -2.114478 -0.509215

81 6 0 -13.426997 -1.976358 -2.544454

82 6 0 -14.496507 -2.515491 -1.835589

83 1 0 -15.492418 -2.517734 0.064439

84 1 0 -13.288127 -2.256072 -3.586410

85 6 0 -13.316674 2.432368 -1.425124

86 1 0 -13.884975 3.286089 -1.800178

87 1 0 -13.931604 1.539510 -1.552837

88 1 0 -12.446299 2.314444 -2.077584

89 6 0 -11.046349 1.033361 2.974847

90 1 0 -11.358175 1.134882 4.017549

91 1 0 -9.973402 1.245140 2.935566

92 1 0 -11.166939 -0.013645 2.697075

93 6 0 -13.412864 5.432087 2.589631

94 1 0 -14.447776 5.681362 2.338698

95 1 0 -12.789897 6.263036 2.240606

96 1 0 -13.331220 5.398039 3.678291

97 6 0 -11.399200 -0.571271 -2.841477

98 1 0 -11.057952 0.426787 -2.566764

99 1 0 -11.717820 -0.533763 -3.886377

100 1 0 -10.523016 -1.225091 -2.793247

101 6 0 -14.074873 -0.833494 1.545388

102 1 0 -14.243931 0.237973 1.668056

103 1 0 -13.242859 -1.101160 2.203560

104 1 0 -14.958583 -1.356071 1.917436

105 6 0 -15.438388 -3.509951 -2.468187

106 1 0 -16.474522 -3.329538 -2.169067

107 1 0 -15.191666 -4.534511 -2.168076

108 1 0 -15.392422 -3.470751 -3.558874

109 6 0 10.986621 1.183843 -2.880957

110 1 0 11.268311 1.345872 -3.924550

111 1 0 9.913831 1.384009 -2.800033

112 1 0 11.122164 0.123929 -2.665692

113 6 0 13.346790 2.362291 1.536126

114 1 0 13.921883 3.197075 1.942171

115 1 0 13.964837 1.465216 1.606403

116 1 0 12.489810 2.210183 2.199128

117 6 0 13.362702 5.558958 -2.325309

118 1 0 13.298626 5.565410 -3.415728

119 1 0 14.388529 5.816911 -2.047935

120 1 0 12.718575 6.364575 -1.955624

121 6 0 11.456876 -0.709508 2.838204

122 1 0 11.111737 0.301617 2.622292

123 1 0 11.795214 -0.726385 3.877419

124 1 0 10.579129 -1.359708 2.772690

125 6 0 14.043683 -0.750261 -1.609172

126 1 0 14.207543 0.326466 -1.682979

127 1 0 13.199174 -0.988586 -2.262724

128 1 0 14.920971 -1.251104 -2.023879

129 6 0 15.491660 -3.617523 2.239207

130 1 0 16.525219 -3.400370 1.955551

131 1 0 15.261715 -4.622227 1.867431

132 1 0 15.444218 -3.655171 3.329825

---------------------------------------------------------------------
